# Tectono-stratigraphic correlations between Northern Evvoia, Skopelos and Alonnisos, and the postulated collision of the Pelagonian carbonate platform with the Paikon forearc basin (Pelagonian–Vardar zones, Internal Hellenides, Greece)

**DOI:** 10.14324/111.444/ucloe.000006

**Published:** 2020-04-24

**Authors:** Rudolph Scherreiks, Marcelle Boudagher-Fadel

**Affiliations:** 1 Geologische Staatssammlung of the Bayerische Staatssammlung für Palaeontologie und Geologie, Luisenstr. 37, 80333 Munich, Germany; 2 Office of the Vice-Provost (Research), University College London, 2 Taviton Street, London WC1H 0BT, UK

**Keywords:** eastern Pelagonia, Paikon collision, ocean floor mélange, shear zone formation, slab break-off, the environment

## Abstract

The Pelagonian stratigraphy of the Internal Hellenides consists of a Permo-Triassic basement and an Upper Triassic and Jurassic carbonate platform formation that has been overthrust by the Eohellenic ophiolite sheet during the Early Cretaceous. Intensive erosion, during the Cretaceous, removed most of the ophiolite and parts of the Jurassic formation. It is hypothesised that uplift and erosion of eastern Pelagonia was triggered by the break-off of the subducted oceanic leading edge of the Pelagonian plate. An investigation of the rocks that succeed the erosional unconformity shows that they constitute a shear-zone that is tectonically overlain by Cretaceous platform carbonates. Geochemical analyses of the shear-zone rocks substantiate that they are of mid-oceanic ridge and island arc provenience. Eastern Pelagonia collided with a Cretaceous carbonate platform, probably the Paikon forearc basin, as the Almopias ocean crust subducted beneath that island–arc complex. The Cretaceous platform, together with a substrate of sheared-off ocean floor mélange, overthrust eastern Pelagonia as subduction continued, and the substrate was dynamically metamorphosed into cataclastic rocks, mylonite, phyllonite and interpreted pseudotachylite. This complex of Cretaceous platform rocks and a brittle-ductile shear-zone-substrate constitute the here named Paikon–Palouki nappe, which was emplaced during Early Palaeocene. The Paikon–Palouki nappe did not reach Evvoia. Seismic tomographic models of the Aegean region apparently depict images of two broken-off ocean-plate-slabs, interpreted as Almopias-lithosphere-slabs. It is concluded that the western Almopias slab began to sink during the Early Cretaceous, while the eastern Almopias slab broke off and sank after the Paikon–Palouki nappe was emplaced in the Early Palaeocene.

## Introduction

**Figure 1:**
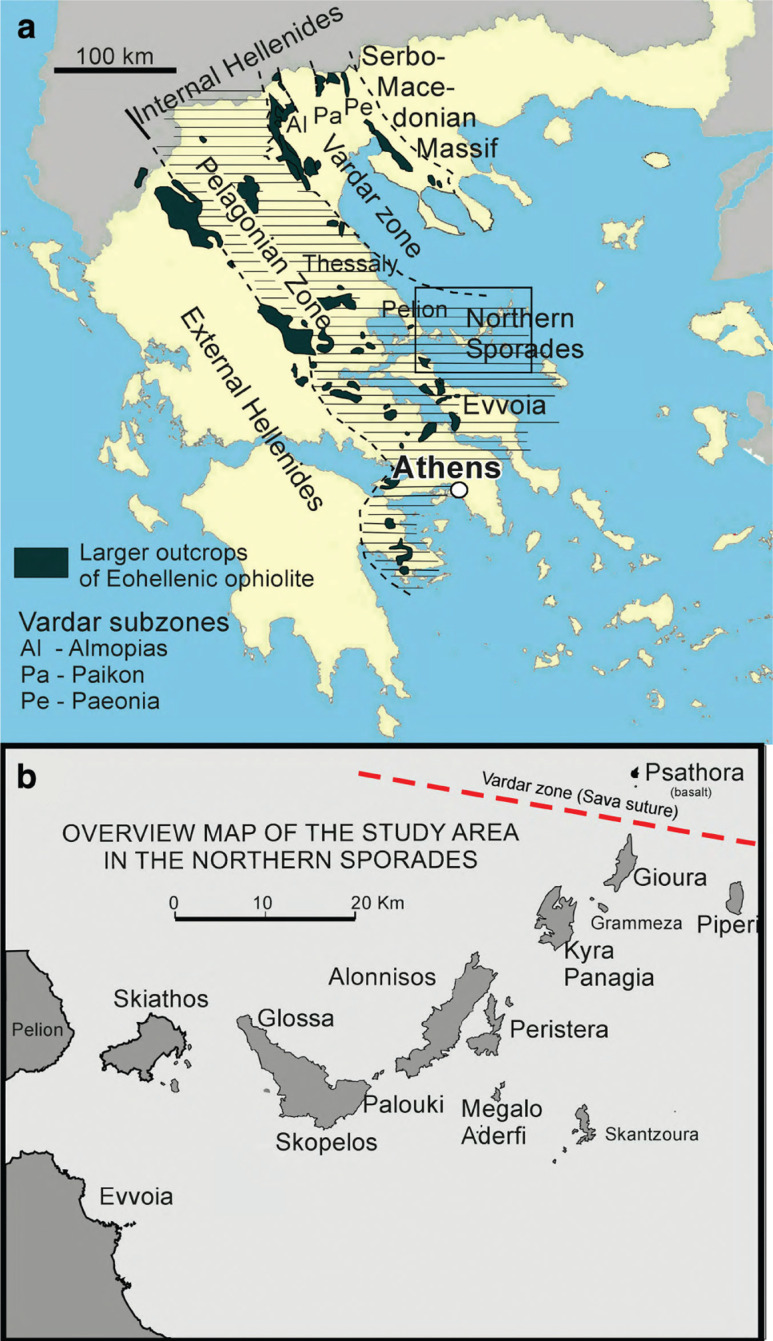
The study area in the Internal Hellenides. (a) The outlined study area extends from northern Evvoia eastwards to Skopelos and Alonnisos and smaller islands of the Sporades. It is in the Pelagonian zone of the Internal Hellenides and borders on the Vardar zone. (b) Overview map of the islands of the study area in the Northern Sporades. Field work was carried out on Evvoia, Skopelos and Alonnisos; reconnaissance field work on Kyra Panagia, Peristera and Megalo Adherfi. The Pelagonian/Vardar-Sava suture is suggested to lie between Gioura and the basalt island of Psathora.

The area described and analysed in this paper is in the easternmost part of the Pelagonian zone of the Internal Hellenides (Fig. 1a). The extensive, over 1-km thick, shallow marine carbonate platform of the Pelagonian zone evolved during the Late Triassic through the Late Jurassic. In the latest Jurassic to earliest Cretaceous, it was buried under an obducted ophiolite sheet, known as an Eohellenic or Western Vardar ophiolite sheet, that derived from the Meliata–Maliac–Vardar (or Neotethyan) oceanic realm [1–5].

The present contribution correlates stratigraphy and tectonics on the eastern margin of the Pelagonian zone, specifically between Alonnisos, Skopelos and northern Evvoia, including observations on the nearby smaller islands of Kira Panagia, Megalo Aderfi and Peristera (Fig. 1b). Special attention is given to a brittle to ductile shear zone, exposed on Alonnisos and Skopelos and the neighbouring islands. We will provide arguments supporting the interpretation that this shear zone is composed of a tectonically sheared mélange that mixes ocean floor and platform carbonate rocks. In our view this tectonic mélange has previously been erroneously interpreted as transgressional conglomerates, meta-turbidites and flysch [6]. On the other hand, this shear zone has also been interpreted as consisting of tectonically deformed erosional relics of metabasites of an oceanic subduction complex, analogous to the Eohellenic ophiolite, or as possible slivers at the base of a Palaeogene nappe [6]. These contradicting viewpoints have motivated the present investigation.

Another controversial point is whether or not Pre-Triassic, Pelagonian basement is at all exposed in northern Skopelos (Glossa area, Fig. 1b), a hypothesis that had previously been positively supported by Papastamatiou [7] and Jacobshagen and Skala [8]. However this exposure was later re-interpreted [9] as being an Eohellenic metabasite. In this the present study, investigations were also carried out in order to shed further light on this controversy.

Field work was carried out during June and September in the years 2016–2018, mainly on Skopelos and Alonnisos with reconnaissance visits to Kira Panagia, Peristera and Megalo Aderfi. Field work was fundamentally aided by previously published geologic maps [6, 9–11], without which this account would not have been possible. Work was further enabled by having new topographic maps of Skopelos and Alonnisos (Nakas Cartography, 1:25,000, 2014 and 2017, Athens, Greece) and an Imray Tetra Nautical Chart of the Northern Sporades 1:183,000, 1995, St. Ives, England). Samples were collected for thin section and geochemical analysis. The geochemical analyses of major, minor and trace elements were performed by Activation Laboratories Ltd. (Ancaster, Ontario, Canada). Boat-field excursions to Peristera and Megalo Aderfi were made possible by ‘Sea Escapes’, Alonnisos, Greece.

The results and interpretations of our investigation are presented below.

## Geological background and previous research

**Figure 2:**
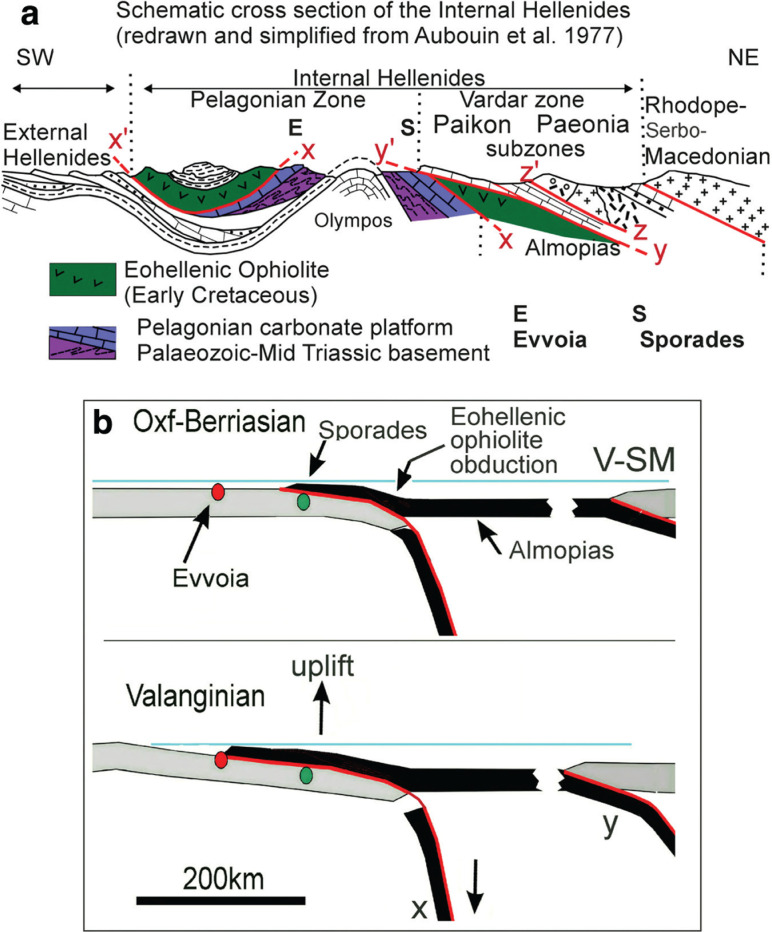
Schematic cross section of the Internal Hellenides (modified after Aubouin et al. [12]; in consideration of: [13, 15], and others in [17]). (a) The zones and sub-zones of the Internal Hellenides are shown and the geotectonic positions of the study areas are indicated. Oceanic crust had obducted onto the Pelagonian zone (x–x′), and, as is discussed in the text, the Paikon and Paeonian thrust faults (y–y′) and (z–z′) do not reach Evvoia. (b) This plate tectonic cartoon of the ophiolite obduction onto the Pelagonian plate is modified from [3]). The Eohellenic ophiolite obduction over the Sporades and Evvoia occurred after the oceanic leading edge of the Pelagonian plate had been subducted. After the ophiolite sheet had reached Evvoia, during Valanginian time, the leading oceanic edge of Pelagonia is supposed to have broken off (slab x), thereby initiating uplifting of the eastern part the Pelagonian plate and erosion on Skopelos and Alonnisos. Convergence and subduction of the eastern Almopias ocean continued (slab y). (V–SM = Vardar–Serbo–Macedonian complex).

The regional tectonic context of the study area is shown in a simplified cross-section through the Hellenides of mainland Greece and across the Olympos window (Fig. 2a), redrawn after Aubouin et al. [12]. This figure essentially indicates that an ophiolite sheet was obducted over eastern Pelagonia during the latest Jurassic to earliest Cretaceous, ‘Eohellenic Phase’ [1, 2], and subsequently overthrust by nappes originating from the Paikon subzone. For greater detail of the actual complexity of the Vardar zone of northern Greece and the Dinarides the reader is referred to Mercier [13, 14], Mercier and Vergely [15], Sharp and Robertson [16], Michail et al. [17], and Schmid et al. [5]. Figure 1a shows the positions of the Vardar subzones. The position of the eastern border of the Pelagonian zone in the area of the Northern Sporades is ambiguous in many previous publications. Here, Pelagonian formations are supposed to extend to the eastern margin of the Sporades, where they border on the Vardar zone (Sava suture: personal communication [5]; Fig. 1b).

**Figure 3:**
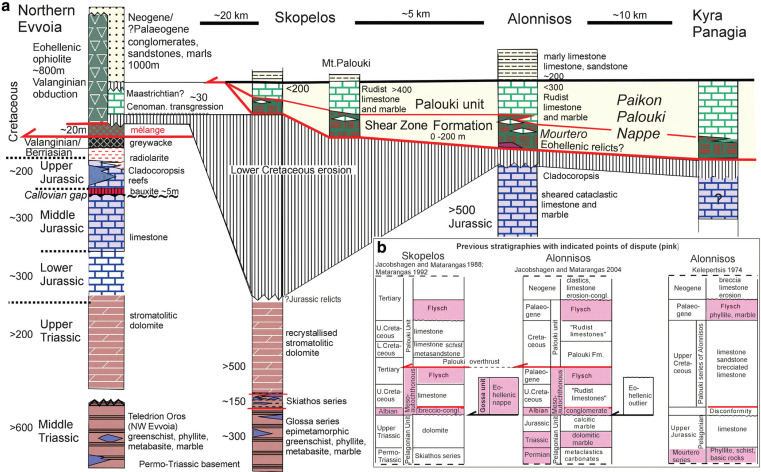
Modified stratigraphy of Alonnisos and Skopelos islands compared to disputed previous stratigraphic interpretations. (a) The relatively continuous stratigraphic column of Evvoia (Permo-Triassic–Cretaceous) correlates well with the basement formations and Triassic dolomite formation of Skopelos, and with the Upper Jurassic reefal facies of Alonnisos. The Jurassic formations have been eroded from Skopelos, with the exception of some tens of metres of earliest Jurassic peritidal dolomite (see text). The Eohellenic ophiolite has been eroded from Skopelos and for the most part from Alonnisos. Note that transgressional Cretaceous is only found on Evvoia. A 0–200 m thick zone of dynamically metamorphosed m¨¦lange containing ocean floor crustal fragments is overlain by the Paikon–Palouki nappe whose stratigraphy indicates tectonic emplacement of this higher nappe occurred during the Early Palaeocene. (b) shows stratigraphic columns of Alonnisos and Skopelos according to previous researchers. The disputed points are underscored in pink colour.
The Mourtero series, equivalent to the shear zone of Alonnisos is actually tectonically situated on top of a lower Cretaceous erosional gap and disconformity, lying directly on the Upper Jurassic.Permian and Triassic formations could not be verified, nor was dolomite found on Alonnisos.The microfauna that had been reported (see text) to indicate an Albian age must be updated to the time-span of Albian-Turonian (see Biostratigraphy).The Glossa unit of NE Skopelos island is considered to represent the Palaeozoic to Permo-Triassic Pelagonian basement of Skopelos island. The ophiolite obducted during the Eohellenic phase was completely eroded on Skopelos island. Eohellenic relicts could not be substantiated to occur on Alonnisos.A Palaeocene-age thrust plane extends over the Pelagonian disconformity over both islands. Post-Jurassic sedimentary rocks were either not deposited or had been eroded in Cretaceous times. The Cretaceous sedimentary rocks of the Palouki nappe and subjacent shear zone are allochthonous.Formations mapped as ¡®flysch¡¯, conglomerates, metaclastics, phyllites and basic rocks by previous workers, are all part of a wider shear-zone that occurs on top of the disconformity and below the base of the Cretaceous carbonate formation of the Palouki nappe.

Geotectonically, the ophiolite of Evvoia, which was obducted during the Eohellenic phase, belongs to the Western Vardar ophiolitic unit that extends through the entire Dinarides and Hellenides [4, 5, 18]. It represents the western part of the northern branch of Neotethys [5] that was obducted, as an oceanic plate, onto the eastern Pelagonian plate. The obduction over Alonnisos and Skopelos occurred during the earliest Cretaceous [6], as the leading oceanic edge of the Pelagonian plate was subducting (Fig. 2b; [3]). An analogous obduction/subduction configuration has previously been suggested (see the general scenario in [4]), showing the West Vardar/Almopias ophiolite being obducted over the Adriatic plate. The post-Eohellenic overthrusts (Y + Z shown in Fig. 2a) did not reach northern Evvoia.

The litho-stratigraphy of Evvoia, Skopelos and Alonnisos has close similarities because during the Triassic until the Early Cretaceous these areas had been part of a continuous carbonate platform. However, in contrast to Evvoia [3], some of the formations of Skopelos and Alonnisos had undergone low grade, sub-greenschist to greenschist metamorphism. Also, Early Cretaceous erosion was much more severe on Skopelos and Alonnisos [8–11] than on Evvoia [19]. During the Early Cretaceous, the ophiolite sheet had been more or less completely removed by erosion on Alonnisos island, and on Skopelos island erosion also removed most of the Jurassic formations, as will be described later.

It has been estimated, based on a tectonic reconstruction [19], that the region eastwards of Evvoia has been uplifted by at least 3000 m since the post-Eohellenic, Early Cretaceous. Uplift may have been caused by buoyancy-rebound of the eastern Pelagonian plate after its subducted oceanic leading edge had broken off (Fig. 2b, [3]; see Discussion below). Eohellenic nappe-relicts may possibly exist on Alonnisos (Eohellenic outlier, [6, 9], but it is highly improbable that *in situ* relicts of Eohellenic ophiolite exist on Skopelos, because intensive erosion has not only removed the ophiolite nappe but also the subjacent Jurassic formation as well. The disputed area of Glossa has been interpreted [6, 9] as being the Eohellenic ophiolite, obducted during the Eohellenic phase (see the discussion below of the Pelagonian Basement). On Evvoia, however, the obducted ophiolite was only partially eroded.

## Disputed and modified stratigraphic logs of Alonnisos and Skopelos

In this paper, new interpretations are presented regarding the stratigraphy of a number of different rock units in the region. The previous stratigraphies are compared to the new stratigraphy (Fig. 3a and b). The modified stratigraphy (Fig. 3a) partially follows previous researchers [7, 20], who recognised that the Pelagonian formations of Skopelos had not been transgressed but had been overthrust by the Cretaceous formations.

Kelepertsis [10] recognised the Jurassic/Cretaceous disconformity but did not recognise the tectonic nature of the rocks that occur between the Jurassic and the allochthonous Cretaceous formations (Fig. 3b). He distinguished the important Palouki series as representing the lateral continuation of the rock unit cropping out on Mt. Palouki of Skopelos island, and he extended the Palouki series from Skopelos to SW Alonnisos (Fig. 3b). Papastamatiou [7] suggested that the Palouki series of Skopelos belonged to the Vardar Zone (Figs. 1a and 2a), a hypothesis that Kelepertsis [10] supported and is, as will be shown below, the interpretation preferred in this study.

The stratigraphic correlations (Fig. 3a) show that the geologic column of Evvoia is more or less continuous from the Permian through the Jurassic until the Lower Cretaceous. The Permo-Triassic basement [21] and the dolomite sequence of Evvoia correlate well with the Glossa and Skiathos series, as well as the Triassic dolomites of Skopelos (Fig. 3a). In contrast, the Triassic is not exposed on Alonnisos island, and has not been verified on Kyra Panagia or Megalo Aderfi either. The Jurassic is, besides a few small enigmatic outcrops, absent from Skopelos, whereas Jurassic constitutes the oldest exposures on Alonnisos, where the reefal facies is similar to that of Evvoia (Fig. 3a). The stratigraphically oldest marbles observed on Kyra Panagia and Megalo Aderfi occur below metabasites and are thought to be of Jurassic age, but no definite verification could be made.

Widespread remnants of the Eohellenic ophiolitic thrust sheet (serpentinite, peridotite, basalt, radiolarite) exist on Evvoia, whereas the inferred ophiolite on the other islands of the study area had been eroded during the Cretaceous, and serpentinite could not be verified. A small *in situ* relict of Eohellenic metabasite is possibly preserved on Alonnisos (see below).

On Evvoia, transgressional conglomerate overlies ophiolite mélange and Tithonian–Berriasian radiolarian cherts [3, 22]. The conglomerate contains serpentinite and radiolarian chert-pebbles, and in turn is overlain by a 30-m limestone succession containing abundant rudist bivalves and foraminifera, which range in age from Coniacian to Maastrichtian [19]. In contrast to this, on the other islands of the study area, *in situ* Cretaceous sediments probably were never deposited (see Discussion below). Instead, the Cretaceous carbonates are allochthonous and overthrust a tectonically mixed brittle-ductile shear-zone (terminology after [23]), composed of a dynamically metamorphosed, oceanic mélange of sericitized basalt and radiolarian chert, which directly overlies the Pelagonian formations.

Occurrences of laterite are widespread on Skopelos [11], in contrast to Alonnisos where laterite is not conspicuous. The re-deposited laterite occurs within joints and karstic fractures in the dolomite formation as well as in cataclastic rocks that can attain thicknesses of up to about 150 m. Primary *in situ* laterite could not be verified. The origin of the primary laterites may have been multi-temporal, considering that the entire eroded ophiolitic thrust sheet and underlying Jurassic carbonates had been their source. On Evvoia, laterite (Bauxite, Fig. 3a) was deposited during the Callovian as well as during the post-Eohellenic period [3, 24].

## The Paikon–Palouki nappe and the brittle-ductile subjacent shear-zone

The Palouki overthrust (Fig. 3b) of Skopelos has been recognised by Jacobshagen and Matarangas [6], who date its emplacement as taking place during the Eocene. According to their interpretation, however, the Palouki overthrust was emplaced over Cretaceous rudist limestones and ‘flysch’ that transgressively overlie the rocks of the Pelagonian (Fig. 3b), an interpretation which is disputed here. Contrary to Matarangas [11], and Jacobshagen and Matarangas [6], we find the Palouki Cretaceous to be underlain by a shear-zone of dynamically metamorphosed, ocean floor basalt and radiolarite, and tectonically intermingled carbonate rocks. The shear-zone rocks range from sheared ocean-floor mélange to sericite schist, greenschist, phyllite and phyllonite (nomenclature following: [23, 25–27]). Recently, Porkoláb et al. [28] determined, via white mica, 40Ar/39Ar dating, an age spectra-dominance of 53–75 Ma in the phyllites of south-eastern Skopelos, indicating that the deformation of the Palouki overthrust took place during latest Cretaceous-Early Eocene time. These results correspond well with our biostratigraphic data shown below.

Numerous occurrences of oceanic rocks on Alonnisos and Skopelos, have been reported previously, including spilitic igneous rocks, serpentinites and metabasalts [10, 20], which were interpreted as Eohellenic metabasites by Matarangas [11] and Jacobshagen and Matarangas [6]. A greenschist of Kira Panagia has been interpreted as a metabasite or as arc-tholeiite basalt-rhyodacite [6, 29]. In this contribution, these rocks are considered to be connected to an all-encompassing tectonic shear zone, that extends from Kira Panagia over Alonnisos, Skopelos, Peristera and Megalo Aderfi, which has not been hitherto recognised. The herein named ‘Paikon-Palouki nappe’, documented below, is composed of allochthonous Cretaceous carbonates that were emplaced together with a subjacent sheared oceanic mélange over the Pelagonian formations of the investigated islands of the Northern Sporades. The 40Ar/39Ar dates [28] and the bio-stratigraphic data (see below) substantiate a latest Cretaceous-Early Eocene emplacement age of the Paikon–Palouki nappe.

### The brittle-ductile shear zone

The nomenclature used here takes into consideration the terminology of ‘shear zones’ [23, 25, 30–33]; among others). ‘Brittle’ refers to fault breccias and cataclasite as in Higgins [25], ‘ductile’ (or plastic) refers to mylonites as in Schmid and Handy [32], whereby neomineralisation and recrystallisation dominate over relict cataclasis. Also, here the terms ‘marble’ and ‘recrystallisation’ do not imply a strictly defined P/T range; they refer to limestones that have been dynamically recrystallised to an extent that most if not all sedimentary fabrics have been obliterated. Metamorphic marble is associated with sericite in the shear zone.

The Cretaceous carbonates of Skopelos and Alonnisos are not in sedimentary contact with their substrate; they tectonically overlie sheared ocean-floor rocks everywhere, verified by geochemical analyses (see below), which in turn overlie Jurassic carbonates on Alonnisos and Triassic dolomite on Skopelos (Fig. 3). The shear zone of Alonnisos and Skopelos has undergone intensive cataclasis and dynamic metamorphism, and low-grade metamorphism characterised by sericitisation of plagioclase feldspars in the oceanic rocks (see below). The shear zone grades upwards from brecciated Triassic dolomite-substrate on Skopelos and brecciated-sheared Jurassic limestones and marble on Alonnisos, into fluxion structured schists (as described in [25, 30]), and further into sericite/chlorite dominated phyllonite (lepidoblastic texture of sericite), and finally, on top, into non-fluxion-structured cataclastic Cretaceous marble and recrystallised limestone (Fig. 3). The sheared, Cretaceous, marbles within the top of the shear zone are composed of alternating, millimetre thin, flat calcite and sericite cleavage planes.

**Figure 4:**
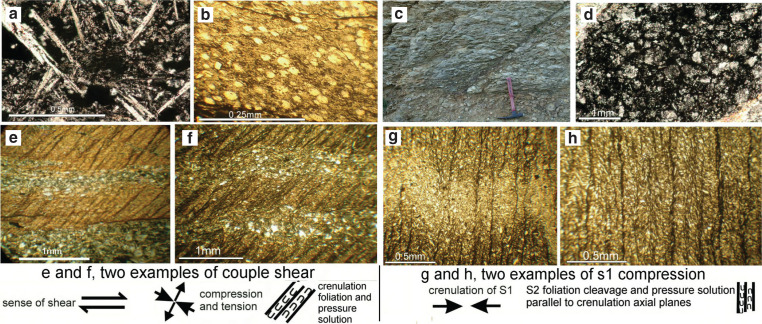
Rocks from within the brittle-ductile shear zone. (a) photomicrographs xpl, of a basalt lithic grain, Alonnisos. Acicular plagioclase in a vesicular basalt. (b) Photomicrograph ppl of slightly deformed radiolarian ghosts in sericitized, fine crystalline quartz, Agios Riginos, Skopelos. (c) Foliated cataclasite resembling a conglomerate, Agios Georgios, Alonnisos, actually a fault rock of the shear zone composed of porphyroclasts aligned parallel to the (+/− horizontal) composite S–C foliation. Secondary shear planes appear to have formed at an angle of about 45¡ã with the composite foliation (s1). (d) Photomicrograph (xpl). This fluxion-structured band of quartz clasts in opaque/isotropic? matter is interpreted as pseudotachylite. The fluxtion-band is bordered by sericite (upper left, lower right). Quartz appears to be disintegrating along the margins, frozen in a flood of opaque matter. (e–f) Photomicrographs ppl, foliated phyllonite. These two examples of shearing-couples show the sense of shear and the crenulation-solution cleavage that formed perpendicular to the compressional-stress component of the shearing couple (examples are described in [34]). (g–h) Photomicrographs ppl, foliated phyllonite. These two examples show development of a new schistosity via a crenulation cleavage (stage 4 in [35]). Examples e–h involve neomineralisation and recrystallisation of sericite.

The shear-zone rocks are composed of porphyroclastic lithic grains of still recognisable basalt, volcanic glass, radiolarian chert and mineral grains of plagioclase (Fig. 4a–c). Secondary minerals are sericite, and quartz having fluid inclusions, chlorite, epidote and chloritoid. The observed lithic grains range in size from a few centimetres to millimetres in rocks that previously have been erroneously identified as Cretaceous conglomerates (Fig. 4c). The components of these rocks underwent tectonic rounding and grain-reduction from the decimetre to centimetre scale to microscopic micron scale, and the matrix material is typically the same as that of the porphyroclasts (a typical phenomenon observed in cataclastic rocks by Higgins [25]). The chlorite, epidote and chloritoid are porphyroblastic minerals, indicating that the temperatures ranged up to about 350°C (see Discussion below). A lithic grain of volcanic glass, inherited from the ocean floor mélange has fine, long needles, of supposed actinolite.

Foliation and rock cleavage is mainly caused by aligned mica [30, 34], or by alternating parallel porphyroclast bands that are crossed obliquely by foliation/shear planes, which may be observed best under the microscope, and of which numerous examples show foliation/cleavage that has been crenulated by compression (Fig. 4e–h). Other examples indicate that the sericite of the foliation plains became reoriented parallel to a secondary shear plane (Fig. 4g, h) (a phenomenon also shown in [35]).

Polyepisodic successions, in which alternating brittle and ductile deformations had taken place, have been seen in numerous thin sections from Alonnisos and Skopelos; typical of shear zones according to Higgins [25]. Likewise, observations of complex overprinted brittle and ductile structures were reported in Meyer et al. [36]. Similar brittle-ductile deformations have been reported as ‘ultracataclastites or pseudotachylites’ by Adams and Trupe [37] in the Alleghanian shear zones. We observe under the microscope that fluxion structured rocks can grade into irregular bands of ‘ultracataclasite’*,* which we interpret as pseudotachylite from descriptions in, for example, Maddock [26] and Higgins [25] (compare also [36]). The supposed pseudotachylite of the study area occurs as millimetre thin, braided sheets of opaque matter, laden with quartz porphyroclasts, which alternate with fluxion structured, aligned sericite, imbedded in interstitial, finer grained matrix, which intrudes adjacent fractures. The quartz grains of the fluxion sheets are serrated, giving the appearance of having been melted on their edges and then frozen in a once fluid medium (Fig. 4d). Pseudotachylite is not uncommon in tectonic thrust and subduction zones from the Alps to Alaska [26, 31, 38, 39]. Higher grade metamorphic minerals other than greenschist facies have not been observed. According to Porkoláb et al. [28] temperatures between 350 and 450°C in the pressure range 1–2 GPa, prevailed during shearing.

## Description of the geologic maps and cross sections of Alonnisos and Skopelos

Geologic mapping was carried out on the islands of Skopelos and Alonnisos and is based on previous geologic maps [10, 11]. Reconnaissance visits to neighbouring islands (Peristera, Kira Panagia and Megalo Aderfi) verified similar geologic formations and structures (Fig. 1b).

### Geology and tectonic sections of Alonnisos

**Figure 5:**
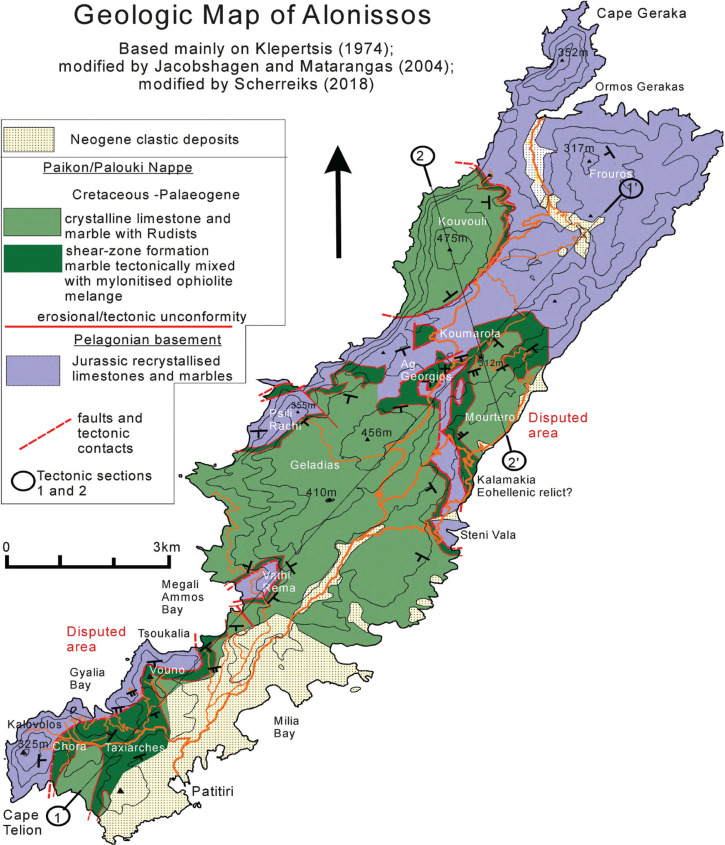
Geologic map of Alonnisos. On Alonnisos, Jurassic crystalline limestones and marbles are the oldest outcropping rocks [10]. After erosion had removed the Eohellenic nappe, the Paikon–Palouki nappe was emplaced during the Palaeocene (see Biostratigraphy). The geologic map of Alonnisos mainly differs from the previous maps in two disputed areas: first, the Jurassic carbonates underlie the shear zone in the area between Cape Telion and Megali Ammos Bay and second, Cretaceous rudist limestones overlie the shear zone in the area between Koumarola and Mourtero.

**Figure 6:**
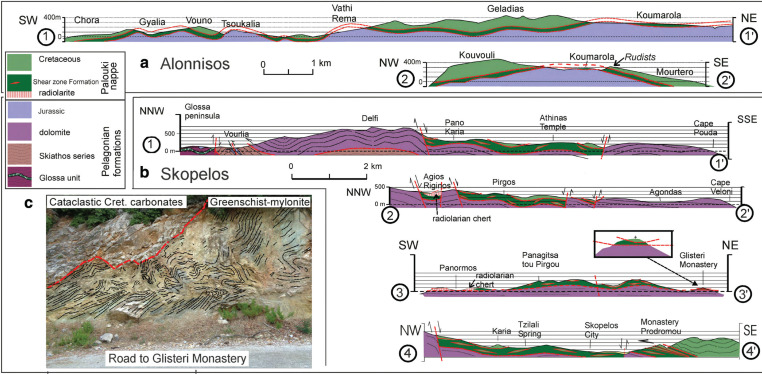
Tectonic sections of Alonnisos and Skopelos. (a1–a2) Sections of Alonnisos show gentle folds without pronounced vergence, however, in the metre range intensive folding had occurred in the *shear-zone* formation. The Cretaceous carbonates are exposed on the highest areas of Alonnisos, for example, Kouvouli and Geladias. Jurassic carbonates underlie the *shear-zone* formation which in turn is overlain by the Cretaceous carbonates that outcrop on the hilltop of Vouno (south of Tsoukalia bay). The section crosses Vati Rema and Vouno, showing folds without pronounced inclination. In the area of Koumarola and Mourtero Rudist bearing Cretaceous marble is exposed along a new road outcrop. Upper Cretaceous overlies the Mourtero unit in a broad antiform. The Jurassic carbonates extend beneath the metabasites at Vati Rema where the correct stratigraphic sequence had already been mapped by Kelepertsis [10]. (b1) The Skopelos section, Glossa–Cape Pouda, shows the stratigraphic succession of the Pelagonian formations between the pre-Upper Triassic basement and the Upper Triassic dolomite. Intra-formational thrusting probably took place between the Triassic dolomite and the Skiathos series, and the basement formations. A distinct NE-SW striking thrust-fault zone, is located between the basement formations and the Upper Triassic dolomite formation, indicating NW vergence. The basement formations probably underlie the dolomite beneath Mt. Delfi of central Skopelos, suggesting that it is about 600 m thick. A graben structure is indicated in the Pano Karia and Athin¨¢s-Temple area, in which mainly the *shear-zone* formation is exposed. (b2) Agios Riginos–Cape Veloni: radiolarian cherts, in which pseudotachylite occurs, is exposed in a graben structure at Agios Riginos where the cherts underlie a large area of brecciated Cretaceous limestones and marbles south of Pirgos. (b3) This section from Panormos to Glisteri Monastery, crosses Panormos bay, where radiolarite and basalt are exposed along the sole of the *shear-zone* formation. The central part of this section shows the shear zone formation overlying the Triassic dolomite. (b4) This section transects the nappe structure of Mt. Palouki, showing that the Mt Palouki Cretaceous carbonate formation together with the shear-zone formation overthrust Upper Triassic dolomite. Some of the best insights of the shear-zone rocks can be seen at Monastery Prodromou. (c) Photograph: road to Glisteri Monastery, showing the outcropping tectonic contact between brecciated Cretaceous crystalline limestones on top of intensively folded, greenschist.

The new geologic map of Alonnisos is shown in Fig. 5, and two tectonic sections of Alonnisos are shown in Fig. 6a. On Alonnisos (Fig. 5), Jurassic crystalline limestones and marbles are the oldest outcropping rocks [10]. The marbles are for the most part coarse crystalline and white, and presumed to be very low grade metamorphic (see comment above). After erosion had removed the Eohellenic nappe, which was emplaced over Pelagonia during latest Jurassic and Early Cretaceous, the Paikon–Palouki nappe was emplaced during the Palaeocene (see Fig. 3a and biostratigraphy). Our geologic map of Alonnisos mainly differs in two critical areas from the previous maps (marked in red, Fig. 5), where the stratigraphic order has been corrected, and is shown in the tectonic sections of Alonnisos (Fig. 6a). Frank [40] and Jacobshagen and Matarangas [6] correctly interpreted the Mourtero series, contrary to Kelepertsis [10] (Fig. 3b) to lie stratigraphically above the Jurassic, and Jacobshagen and Matarangas [6] correctly concluded that the Mourtero unit, as a whole, had overthrust the Jurassic marbles. These observations are important for the present tectono-stratigraphic interpretation.

### The Pelagonian basement of Skopelos

**Figure 7:**
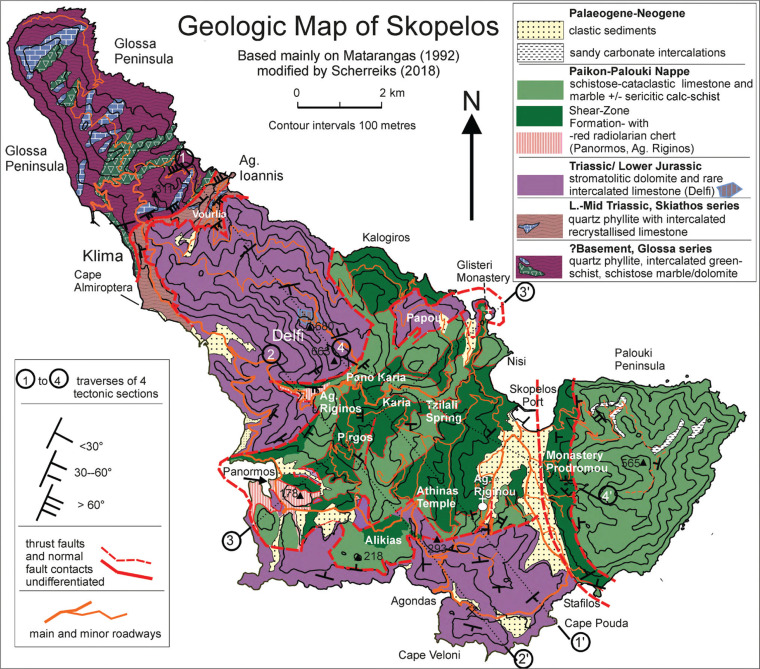
Geologic map of Skopelos. The Pelagonian formations extend stratigraphically from the pre-Middle Triassic basement (Glossa and Skiathos series) to Upper Triassic/?Lower Jurassic dolomites (Mt. Delfi). The Triassic dolomite is brecciated and permeated by faults in all areas where it overlies the basement. The Eohellenic ophiolite that once covered the Pelagonian formations has been removed by erosion. A tectonically emplaced shear zone formation overlies the dolomite formation and is overlain by allochthonous Cretaceous limestones and marbles of the *Paikon–Palouki nappe*. These formations occur in the southern part of Skopelos and also build the Palouki Peninsula. The position and traces of the tectonic sections 1–4 are indicated on the map and are shown in Fig. 6b.

Previous authors considered the Glossa-series (Figs 3a, b and 7) to represent the Permo-Triassic, Pelagonian basement of Skopelos [7, 8]. However, this has been contradicted and re-interpreted by Matarangas and Jacobshagen [9] and Matarangas [11], who maintain that the rock assemblage of the Glossa unit is identical to ‘Eohellenic ophiolite outliers on Alonnisos’ (Fig. 3b). They argue that the Glossa unit has been thrust from the northeast over the Upper Triassic formations of Skopelos. However, the fault-trace that crosses the island over mountainous terrain from Klima to Agios Ioannis (Fig. 7) has been incorrectly interpreted as a south-eastward-vergent, low-angle thrust fault [9]. The fault trace and its intersection with topography actually indicate a low angle SE dipping (30°) thrust fault in the Klima area, which shows that the Triassic dolomite had been thrust north-westwards over the Skiathos- and Glossa-series (see Fig. 6b1 and Fig. 7). The Skiathos- and the Glossa-series are low-grade, para-metamorphic rocks with intercalations of metabasites, similar to the basement of Evvoia, consisting of quartz-phyllites and chlorite-sericite-schists intercalated with, micaceous quartzite, calc-schist and sheared marble (Katsikatsos et al., 1984). On Skopelos (Fig. 7), Upper Triassic dolomite overlies the Skiathos series concordantly, but the dolomite is tectonically brecciated and permeated by faults in all areas where it borders on the subjacent basement suggesting that it overlies a fault zone (Vourlia in Fig. 6b1 and Fig. 7). In agreement with the older views [7, 8], the formations of the Glossa peninsula are considered to belong to the Permo-Triassic basement of Skopelos (see further arguments in the Discussion below). Most important is that Palaeozoic, Variscan protolith ages (>250 Ma) have been verified, based on 40Ar/39Ar dating [28], although the depositional age of the Glossa unit has not been determined.

Porkoláb et al. [28] have dated white mica 40Ar/39Ar ages, in the quartz phyllites of the Glossa unit, showing that the mica underwent low grade heating during the Early Cretaceous. These authors reiterate the stratigraphic interpretation of Matarangas and Jacobshagen [9] and Matarangas [11], assuming that the Glossa unit is of Eohellenic provenience. However, the Early Cretaceous white-mica metamorphosis cannot be taken as the age of the para-metamorphic Glossa unit, which is, according to our interpretation, much older. The findings of Porkoláb et al. [28] affirm that the basement of the Pelagonian carbonate platform was affected by Variscan as well as Eohellenic thermal metamorphism, of which the latter occurred as the Eohellenic sheet was obducted over both the Jurassic–Triassic platform, and the basement of the Pelagonian plate during the Early Cretaceous.

### Description of the tectonic sections

The NW-SE tectonic section through Skopelos (Fig. 6b1) shows the interpreted geology from the Pelagonian basement of Glossa through the tectonically disturbed basement-zone around Vourlia and the moderately folded Triassic dolomite formations to the area of Cape Pouda at the SE end of the section. By way of tectonic interpolation, it is suggested that the Skiathos series and basement lies about 600 m below the dolomite of Mt. Delfi. The central area of Skopelos is dominated by typical Pelagonian, Upper Triassic dolomite. Close to the vicinity of Mt. Delfi (Fig. 7) calcareous intercalations occur in the dolomite, that are similar to the transition zone from Upper Triassic to Lower Jurassic, observed on Evvoia, where the dolomite formation extends stratigraphically into the Lower Jurassic and alternates increasingly with limestones in the centimetre to metre range. This suggests that lowest, Lower Jurassic may still be preserved on Skopelos. Flysch-facies could not be verified in the Delfi area as has been previously recorded [11]. Otherwise, Jurassic carbonates have been removed by erosion on Skopelos. It is noteworthy that neither dolomite-marble nor sericite could be observed in the Triassic stromatolitic dolomites that lie beneath the shear-zone. Similar to Evvoia, the tectonic deformations of the Pelagonian carbonates apparently took place below the sub greenschist-facies temperature range of around 200–250°C (compare [3]; also, [30]).

The sections (Fig. 6a,b) show that the Jurassic and Triassic Pelagonian carbonates are overlain by the brittle-ductile shear-zone (defined above) which in turn is overlain by Upper Cretaceous carbonates of the Palouki unit. Together, the Cretaceous Palouki carbonates and underlying shear-zone comprise the Paikon–Palouki nappe, which is postulated in the Discussion to have had its origin in the Paikon subzone (Fig. 2). The sections (Fig. 6a, b) show gentle folds, on the 100-m scale, both on Skopelos and Alonnisos. The large-scale folds do not have a pronounced or apparent preferred vergence and as Matarangas [11] reports, there are three main fold-trends: NNW-SSE, NNE-SSW, and ENE-WSW. As Matarangas noted, in the metre scale, there are pronounced inclined and recumbent folds, which have been encountered during the present investigation in the tectonic shear zone, and their axes trend parallel to the thrust-planes of the major faults. These thrust directions do not correspond to the usual vergence of emplacement of the Eohellenic nappe that one observes in Evvoia or from the general thrust directions (top toward the SW) of the west Vardar Zone (Almopias) ophiolite (Fig. 2 and [3]). The observations of three (*contradicting*) main fold trends [11] and major W to NW-vergent thrust faults, suggest that the area of Skopelos has been progressively rotated clockwise during episodes of tectonic deformation. However, the suggested rotation that had probably taken place in the Sporades is not addressed in this presentation because this subject, although important, goes far beyond the scope of the present theme, and palaeomagnetic data is not available (but we note that rotations affecting Pelagonia and Evvoia have been recognised and previously considered in [19]).

### The allochthonous Cretaceous of the Palouki unit and subjacent shear zone

Contrary to Matarangas [11], the Cretaceous formations of Skopelos are not in sedimentary contact with the subjacent Pelagonian dolomite formation. For example, in the Panormos and Agios Reginos areas radiolarian chert and metabasite occur in tectonic contact on top of the Triassic dolomite (Fig. 6b2 and b3). Transgressional conglomerate could not be verified. The Paikon–Palouki nappe was thrust over the islands following the time of erosion of the Eohellenic ophiolite and underlying Jurassic formation. Sheared oceanic rocks overlie the Triassic dolomite of Skopelos and the Jurassic carbonate succession of Alonnisos. The hitherto interpreted ‘flysch’ of Alonnisos and Skopelos (Fig. 3b) is not a sedimentary formation but is part of the brittle-ductile shear zone that occurs below the Cretaceous carbonate rocks. Cretaceous rocks are pervasively intermingled with the schists in an alternating brittle-ductile transition zone that lies on top of the post-Eohellenic erosional disconformity (Figs 3a, 6a and b). An excellent outcrop that shows the stratigraphic sequence of Cretaceous carbonate rocks on top of mylonitic greenschists may be observed in the Glisteri-Skopelos Bay area (Fig. 6c). The shear zone attains a thickness of about 100 m on Alonnisos (Figs 5 and 6a), Morterio, Taxiarchis and Skopelos town (Fig. 7), but attains more than 200 m in the Palouki fault-zone, and in the areas of Pirgos and Kalogiros (Fig. 7). The fluctuating thickness of the shear zone, swelling to hundreds of metres and diminishing to almost zero, probably is a result of imbrication and accretion, and, respectively, due to tectonic off-scraping as the Paikon–Palouki nappe was transported and emplaced. Good exposures of the shear zone can also be seen near Monastery Prodromos on the northern foot of Mt. Polouki (section 4 of Fig. 6b). The tectonic sections (Fig. 6b) also show interpreted graben structures, for example, between Pano Karia and Athinás Temple (Fig. 6b1) and half-graben structures which post date the emplacement of the Paikon-Palouki nappe. Whether or not these post-compressional tectonic structures can be related to those that exist below sea level to the north and north-west of the study area [41] has not been followed in this presentation.

### Biostratigraphy

The biostratigraphic data presented below substantiates the ages of the carbonate rocks that occur above and below the Paikon–Palouki nappe and that occur mixed into the shear zone.

### Alonnisos

An Upper Jurassic age has been determined previously by Kelepertsis [10] and is substantiated in this contribution with a *Cladocoropsis* find in the area of Mourtero (GPS N39°12.517′; E23°55.562′). The Upper Cretaceous formation of Alonnisos has been dated with rudists [10], which could also be substantiated (road outcrop near trigonometric point 312 m north of Mourtero, Fig. 5).

### Skopelos

In the Palouki tectonic zone, near the Prodromou Monastery, Matarangas [11] reported finding *Actinoporella* *podolica* (A. Alth), in thin-bedded limestone (actually sheared and foliated sericitic marble), with intercalations of phyllites and ‘metasandstones’. This reported foraminifer indicates Early Cretaceous Berriasian–Barremian age. Above the latter sheared limestone zone, rudist limestones and marbles occur along the road leading through the Upper Cretaceous succession of Mt. Palouki. The age range, encountered in Mt. Palouki, extending from Early to Late Cretaceous, without intervening Eohellenic ophiolite substantiates its allochthonous provenience.

Within the shear zone, north of Palouki, the mixed-in carbonate rocks show a wide range of ages. North of Mt. Palouki, Matarangas [11], found microfossils in cementing material or matrix of a conglomerate 30 cm above Triassic dolomites in the Agios Riginou Monastery area (church symbol east of Athenás Temple, Fig. 6b1 and Fig. 7). The microfossils presented by Matarangas are not specific for the Albian, as he had assumed, but have a wide stratigraphic range from at least Albian to Turonian. Table 1 shows the new names and updated age-ranges.

**Table 1.** Observed and identified fossils.

A)	Agios Riginou [11] updated BouDagher-Fadel	
	•	*Valvulammina picardi* Henson
	•	Updated to: *Nezzazatinella picardi (Henson), Albian to Santonian*
	•	Zezzazata *convexa* Fleury (assumed Albian age)
	•	Species not recognised
	•	*Discocyclina schlumberger¨¬* Munier-Chalmas
	•	Updated to: *Dicyclina schlumbergeri* Munier-Chalmas,
		Albian to Santonian
B)	Agios Riginou, side road to Monastery, 5 km S of Skopelos (ident. BouDagher-Fadel)	
	•	*Rotorbinella* sp.,
	•	*Orbitoides* sp.,
	•	*Lithocodium* sp.
	•	recrystallised algae
		late Santonian to Maastrichtian
C)	SE of Agios Riginou, limestone at hairpin turn of main road (ident. BouDagher-Fadel)	
	•	*Kathina* sp.,
	•	*Daviesina* sp.,
	•	*Lockhartia* sp.
	•	Barnacle species
	•	recrystallised algae
		Early Paleocene
D)	Area south of Panormos Bay (ident. BouDagher-Fadel)	
	•	rudists
	•	*Orbitoides* sp. (fragment)
	•	*Rotorbinella* sp.,
	•	*Lithocodium aggregatum*
	•	recrystallised algae
late Santonian to Maastrichtian		

In the same area of Agios Riginou Monastery, the present authors investigated limestones containing recrystallised rudists, which disclosed, in thin section, foraminifera and algae of Maastrichtian age (Table 1). The location is the side road to Agios Riginou (Fig. 7), south of Skopelos Port. The limestone-wackestone contains recrystallised rudists from the families Radiolitidae and Hippuritidae, indicating a shallow marine, reefal facies [42, 43].

Southeast of Agios Riginou, stratified fossiliferous limestone occurs on top of reddened lateritic breccia (Table 1C) (hairpin turn on the main road south of Skopelos town Fig. 7). The lateritic, cataclastic limestone lies on top of Triassic dolomitic rocks, and it contains relicts of molluscs and smaller shelly organisms. Thin sections of the wackestone disclosed a shallow reefal facies with recrystallised algae and benthic foraminifera indicating Palaeocene age [42, 43].

In numerous areas of Skopelos, Upper Cretaceous age has previously been ascertained [11]. In the area south of Panormos Bay (Fig. 7), where Matarangas reported microfossils indicating Cenomanian age, the present authors can contribute findings of shallow reefal wackestones, containing fossils (Table 1D) which expand the age of the Cretaceous in the southern Panormos area from Cenomanian to Late Santonian to Maastrichtian/Early Palaeocene [42, 43]. The stratigraphy of these limestones is tectonically disrupted, and the rocks are mingled into the shear-zone formation.

The stratigraphic range of *Lithocodium aggregatum* Elliott, known so far, is Tithonian–Coniacian, but there are rare records of *Lithocodium aggregatum* Elliott in the Late Cretaceous (e.g. [44, 45]). The presence of a fragment of *Orbitoides* sp. indicates an age of late Santonian to Maastrichtian; *Rotorbinella* sp. to Late Cretaceous–Early Palaeocene. Therefore, as it appears, the limestone/marble formations of central Skopelos, north of Mt. Palouki, range in age from Late Cenomanian to Maastrichtian and may extend into Palaeocene.

## Geochemistry of the shear zone

Analyses of major, minor and trace elements were carried out for 22 samples from the shear-zone described above from Alonnisos, Skopelos and Kira Panagia (Table 2a and b). For reasons of comparison, three samples were analysed from the Glossa area, and three from Evvoia: one mid-ocean ridge basalt (MORB), one serpentinite and one peridotite. The analyses were made with the intention of distinguishing the regime or regimes from which the shear-zone rocks possibly originated (following [46–52]).

**Table 2.** Geochemistry.

A																				
	Si0_2_	A1203	Fe203*	MnO	MgO	CaO	Na20	K20	Ti02	P205	LOI	Total	Sc	V	Sr	Y	Zr	Cr	Co	Ni
A1/1	58.29	18.12	7.98	0.055	4.03	0.38	1.08	3.42	0.836	0.11	6.36	100.7	19	139	44	28	184	250	19	100
A2/2	66.12	14.36	6.12	0.034	2.58	0.36	1.15	2.91	0.734	0.13	5.56	100.1	16	125	48	27	185	240	13	90
A3/14	73.19	10.08	7.50	0.083	2.90	0.27	1.88	0.82	0.608	0.04	2.69	100.1	17	201	74	29	83	130	17	30
A4/17	57.29	14.41	12.66	0.129	6.53	0.05	0.09	3.31	0.914	<0.01	4.86	100.2	25	161	5	28	147	1620	74	1070
A5/29	78.56	7.60	4.14	0.141	2.86	0.18	1.26	0.85	0.563	0.08	2 84	99.07	8	49	28	22	175	540	12	100
A6/31	61.12	17.05	7.16	0.086	3.73	0.29	1.14	3.38	0.756	0.13	5.18	100.0	18	140	54	25	191	240	18	110
A7/3.1	47.06	14.20	9.76	0.184	8.68	10.21	1.01	0.54	0.436	0.03	8.07	100.2	43	214	307	10	28	490	39	110
A7/3.2	50.76	15.71	11.17	0.128	8.56	3.44	3.30	2.13	0.587	0.03	4.12	99.93	47	231	118	12	33	260	44	70
A10/38	74.03	10.71	5.47	0.048	2.84	0.14	1.42	1.61	0.532	0.01	3.11	99.93	18	132	24	20	104	110	12	20
A11/39	57.55	13.42	8.02	0.158	7.51	3.33	4.93	0.04	0.441	0.02	5.41	100.8	39	210	111	8	22	530	30	110
A12/40	64.89	13.85	7.88	0.069	4.25	0.15	2.53	1.07	0.865	0.04	3.50	98.89	22	171	104	30	99	170	23	50
A13/41	73.85	8.15	7.02	0.080	4.24	0,42	0.59	0.16	0.527	0.03	4.05	99.11	13	122	20	31	50	80	29	<20
A5/17	46.78	15.40	11.53	0.162	6.52	8.41	3.12	0.24	1.908	0.24	6.24	100.5	41	314	180	37	143	240	42	110
A8/17	57.23	15.94	10.94	0.075	3.33	0.25	3.15	1.99	1.248	0.09	3.86	98.10	33	190	59	32	100	110	28	40
S1/46	63.16	16.57	6.65	0.193	2.88	0.45	1.07	3.37	0.787	0.16	4.97	100.3	17	138	65	24	194	220	27	90
S2/47	67.54	14.16	6.14	0.060	2.57	0.51	1.57	2.31	0.730	0.14	4.90	100.6	13	101	72	23	187	130	14	50
S6/55	49.22	19.17	10.84	0.042	6.5	1.48	1.81	3.23	0.924	0.17	7.32	100.7	21	167	68	37	199	270	29	230
S11/62	62.48	16.57	6.13	0.070	2.03	0.87	1.27	4.35	0.837	0.13	5.85	100.6	16	135	27	24	227	140	18	60
S7/17	19.12	5.99	2.58	0.092	1.25	37.36	0.68	1.30	0.246	0.05	31.49	100.2	7	49	877	11	52	60	5	40
G1	55.46	14.64	6.34	0.072	3.56	6.36	1.85	2.69	0.694	0.10	7.29	99.05	16	135	208	19.9	132	180	20	100
G17	47.14	15.20	10.43	0.175	8.68	9.39	2.09	1.08	1.409	0.14	4.07	99.79	41	274	136	32.1	87	350	43	90
G41	51.66	21.17	8.54	0.059	3.50	0.31	0.68	6.39	1.058	0.17	5.59	99.13	22	178	15	34.7	221	270	21	140
A1	55.42	16.18	9.92	0.111	5.43	1.03	6.11	0.03	0.611	0.04	4.83	99.71	46	338	103	17	28	<20	34	20
A8	61.38	16.66	7.44	0.077	4.11	0.32	1.25	2.95	0.815	0.1	4.43	99.55	18	137	58	23	162	210	17	110
S5	47.33	15.64	12.26	0.160	7.43	5.78	3.64	0.28	1.959	0.22	5.11	99.81	41	361	32	38	153	240	42	110
MORB	53.08	13.19	7.30	0.226	10.44	2.80	2.56	1.37	1.147	0.15	6.46	98.74	37	212	76	20	80	240	35	110
Serp	36.86	0.59	8.24 0.089		40.71	0.20	0.01	<0.01	0.009	<0.01	11.94	98.66	8	31	2	<1	<2	2440	112	2500
Perid	42.13	1.08	8.91 0.130		45.25	1.32	0.03	0.01	0.010	<0.01	0.20	98.67	12	47	<2	<1	<2	3040	112	2440

B																	
	Nb	La	Ce	Pr	Nd	Sm	Eu	Gd	Tb	Dy	Ho	Er	Tm	Yb	Lu	Th	U
A1/1	12	38.1	63.9	9.06	3S.6	7.2	1.40	6.3	1.0	5.6	1.1	3.2	0.46	3.0	0.44	12.2	1.8
A2/2	10	32.7	68.0	7.98	31.3	6.4	1.36	5.9	1.0	5.7	1.1	3.2	0.45	3.0	0.45	11.5	2.1
A3/14	<1	7.5	27.3	2.85	12.7	3.6	1.18	4.3	0.8	5.3	1.1	3.3	0.50	3.4	0.46	1.2	0.5
A4/17	15	11.7	21.5	3.11	12.8	3.7	0.99	4.7	0.9	5.5	1.1	3.3	0.47	3.1	0.46	7.7	2.1
A5/29	5	25.9	47.5	6.48	26.0	5.2	0.87	4.5	0.7	4.3	0.9	2.8	0.43	2.8	0.43	6.6	2.0
A6/31	11	31.5	58.1	7.50	28.8	5.9	1.15	5.2	0.8	4.8	1.0	3.0	0.43	2.8	0.42	12.0	2.2
A7/3.1	<1	1.3	4.6	0.61	3.3	1.2	0.46	1.6	0.3	2.0	0.4	1.4	0.22	1.4	0.20	0.5	0.2
A7/3.2	<1	2.7	7.0	1.09	5.5	1.8	0.65	2.4	0.4	2.9	0.6	1.7	0.24	1.5	0.22	0.5	0.2
A10/38	<1	8.0	23.3	2.94	13.4	3.4	0.91	3.3	0.6	3.6	0.8	2.4	0.38	2.4	0.37	2.0	0.8
A11/39	<1	1.3	3.6	0.52	2.8	1.0	0.33	1.5	0.3	1.7	0.3	1.0	0.16	1.1	0.16	0.3	0.1
A12/40	1	9.3	19.8	3.65	17.4	5.2	1.49	5.7	1.0	6.1	1.3	3.8	0.56	3.7	0.60	1.4	0.5
A13/41	<1	6.1	14.9	2.27	11.0	3.6	1.01	4.6	0.8	5.7	1.1	3.3	0.47	3.1	0.46	0.9	0.5
A5/17	3	5.1	15.5	2.60	14.2	4.6	1.55	6.3	1.1	7.0	1.4	4.3	0.61	4.0	0.54	0.2	0.1
A8/17	<1	9.9	20.6	3.21	15.3	4.7	1.50	5.2	0.9	6.4	1.3	3.8	0.58	3.8	0.56	1.2	0.4
S1/46	11	36.6	69.3	8.70	33.1	6.6	1.31	5.7	0.9	5.0	1.0	2.8	0.43	2.8	0.43	12.1	2.7
S2/47	7	26.3	54.5	6.64	26.0	5.4	0.99	4.7	0.8	4.4	0.9	2.7	0.39	2.5	0.38	9.5	1.7
S6/55	14	31.5	65.5	7.74	30.0	6.2	1.21	6.3	1.1	6.7	1.3	3.6	0.49	3.0	0.45	13.6	2.3
S11/62	10	31.0	64.1	7.57	29.6	5.8	1.06	4.9	0.8	4.7	0.9	2.8	0.41	2.7	0.42	11.3	2.2
S7/17	4	12.5	21.7	2.72	10.9	2.2	0.50	2.0	0.3	1.8	0.4	1.2	0.18	1.2	0.17	3.9	0.7
G1	12.2	30.4	60.0	6.71	25.8	5.18	0.930	4.41	0.62	3.58	0.71	2.07	0.312	2.12	0.321	9.58	1.93
G17	1.9	3.65	10.6	1.91	10.6	3.60	1.43	4.82	0.86	5.57	1.14	3.37	0.517	3.31	0.484	0.10	0.18
G41	19.8	47.7	80.0	10.9	42.4	9.21	1.64	7.33	1.09	6.65	1.28	3.51	0.541	3.36	0.524	15.0	2.91
A1	<1	1.9	5.2	0.66	3.2	1.3	0.30	2.0	0.4	2.6	0.6	1.9	0.29	2.0	0.32	0.7	0.1
A8	12	23.0	49.1	5.54	20.8	4.5	0.97	4.1	0.7	4.3	0.8	2.4	0.37	2.3	0.34	12.2	1.5
S5	4	5.4	15.6	2.72	14.3	4.6	1.44	6.7	1.1	7.2	1.5	4.3	0.66	4.1	0.61	0.3	<0.1
MORB	8	10.4	27.4	2.97	12.6	3.3	0.94	3.9	0.7	4.0	0.8	2.40	0.35	2.2	0.32	2.5	0.5
Serp	<1	<0.1	<0.1	<0.05	<0.1	<0.1	<0.05	<0.1	<0.1	<0.1	<0.1	<0.1	<0.05	<0.1	<0.01	<0.1	<0.1
Perid	<1	<0.1	<0.1	<0.05	<0.1	<0.1	<0.05	<0.1	<0.1	<0.1	<0.1	<0.1	<0.05	<0.1	<0.01	<0.1	<0.1

Analyses performed by Actlabs, Canada, with fusion inductively coupled plasma (ICP) atomic emission spectroscopy) and fusion mass spectrometry (MS).MORB, mid-ocean ridge basalts; Perid, peridotite; Serp, serpentinite; SiO_2_.

**Figure 8:**
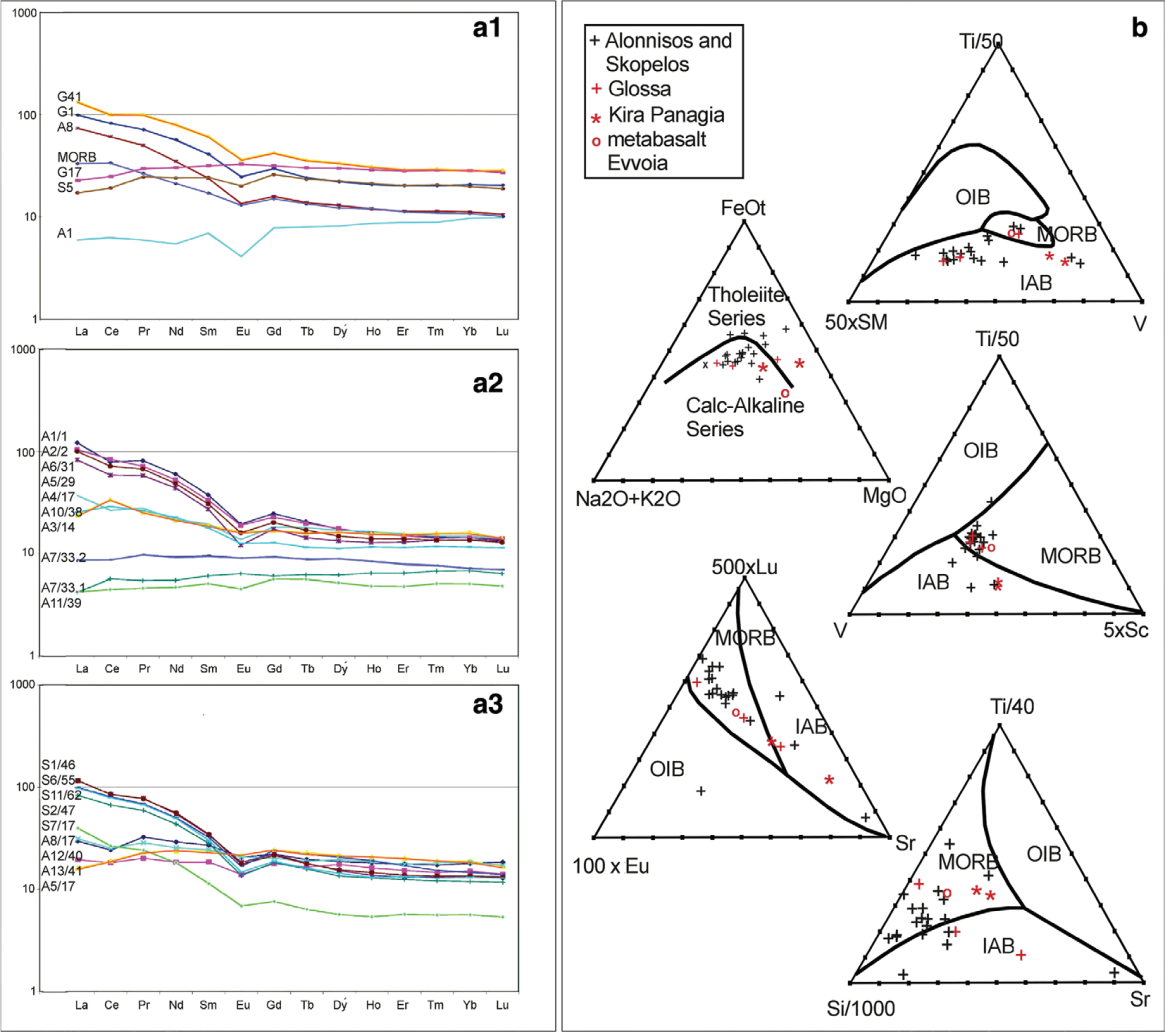
REE plots against chondrite (ppm) and discrimination diagrams. (a1–a3) The REE plots are of rocks from the *shear-zone* formation with the exception of a MORB and peridotite from Evvoia and three samples from the Glossa area (G41, G1, G17). The plots indicate that the samples from the shear zone are of basaltic origin, mostly having negative Eu anomalies. There are mainly two groups: those with relative light REE enrichment and those with relative light REE depletion. Contamination occurred because of tectonic mixing with limestones, for example, S7/17, a calc-schist. (The plots, a1–a3, of the individual samples are coincidentally arranged in the order that they came from the laboratory, the sample codes are on the left. Their order does not correspond to the geochemistry Table 2A and 2B.) (b) Discrimination diagrams compiled from samples of Table 2A and 2B. The AFM diagram shows that the samples correspond to the calc-alkaline series with few exceptions. The discrimination diagrams indicate MORB provenience and IAB affinities (see text).

The geochemical analyses substantiate their oceanic origins, which is readily recognised in thin section especially in the porphyroclastic rocks, showing mixtures of basalt, volcanic glass, radiolarian chert and mineral grains of plagioclase (Fig. 4a, b). However, many of the rocks of the shear-zone are not obviously of oceanic origin: for example, the phyllonites, consisting of concentrations of white mica, quartz and chlorite. In addition, the geochemistry of the oceanic origins is masked by admixtures of carbonate rocks, stemming from the Cretaceous formation on top of the shear zone formation (Table 2a, e.g. samples A7/3.1, S7/17). The rare earth elements (REE) analyses of these rocks display plots that correspond to MORB and Island arc basalt (IAB) patterns (Fig. 8a1, a3). It is very surprising that even the analysed phyllonites in which the original minerals had undergone retrograde metamorphic, hydrothermal metasomatism (see Discussion below), and mylonitisation still display characteristic basalt–REE-plots (e.g. sample A6/31 in Fig. 8).

The REE plots (Fig. 8a), in all shear-zone rocks, display patterns typical of basalts. Two main groups can be distinguished: those having enriched light REE (LREE), as opposed to those having flat plots displaying LREE depletion. Based on numerous observations (e.g. [46]), IAB is generally more variable than MORB, which is characterised by LREE depleted, displaying flat curve patterns. IAB patterns are commonly flat but may be LREE enriched with variable Eu anomalies ([50], e.g. New Britain, Aleutians, Sunda). Most of the REE examples of the study area have negative europium (Eu) anomalies, and only in a few is an Eu anomaly absent (see Discussion).

The REE plots (Fig. 8a) and discrimination diagrams (Fig. 8b) (after [53]) show that the samples are either distributed in the MORB or IAB fields. The AFM diagram shows that the rocks are mainly of the calc-alkaline series and fewer fall into the tholeiitic series field. These results substantiate that the shear-zone rocks are metabasites, mostly of a MORB origin but a few indicating IAB provenience.

Peridotite and serpentinite from Evvoia have been analysed in order to compare their trace-element contents with the rocks of the shear-zone formation. Only one sample from Alonnisos (Table 2A sample A4/17) has chromium (Cr) and nickel (Ni) contents comparable to serpentinite or peridotite, which probably indicates that ultrabasic porphyroclasts probably occur in the shear zone formation, although rarely. Nevertheless, this sample has a basalt REE plot (Fig. 8a2).

## Discussion

### Pelagonian basement

Previously, it has been maintained that the rock assemblage of the Glossa unit is identical to ‘Eohellenic outliers’ [9, 11] and affirmed with two arguments, each of which is disputed in this presentation:

1. According to the cited authors, the Glossa series is not of Permo-Triassic age because, as they claim, it is completely different from other Pelagonian pre-Mesozoic basements. They used as an example the crystalline complex of Thessaly, where a metaclastic series is overlain by a thick sequence of Pelagonian marbles. This is not, however, the case in nearby Evvoia where the Upper Triassic dolomites do not overlie a metaclastic series, but on the contrary they overlie a thick (> 600 m), Permian–Middle Triassic succession of phyllites and schists with intercalated Prasinite-greenstones (metabasites) and fossiliferous limestones (Katsikatsos et al., 1984) [21], that, except for the fossils, resemble the Glossa sequence excellently.
2. Matarangas and Jacobshagen (1988) [9, 11] base their idea of an Eohellenic origin of the Glossa unit on geochemical studies of metabasites in the Glossa series, which indicate MORB affinities of a geotectonic setting comparable to a marginal or an oceanic basin. Metabasites, however, are not specific only for the Eohellenic nappe: metabasites also occur in the Middle Triassic basement-succession of Evvoia and have likewise been interpreted as having been derived from submarine extrusions of basic igneous rocks (Katsikatsos et al., 1984) [21]. For this reason, Glossa green-schists have been analysed geochemically [List 2, REE plots] (Fig. 8a) and discrimination diagrams have been constructed (Fig. 8b); they substantiate MORB/IAB and calc-alkaline basalt affinities for the Glossa unit. Geochemical data cannot be used to distinguish pre-Upper Triassic basement-metabasite from Eohellenic metabasite. The analysed metabasites of Glossa (Table 2, G41 and G1) are partly in the IAB field and have negative Eu anomalies, whereas G17 has a flat MORB pattern without an Eu anomaly. The ‘IAB’ types can be interpreted as within plate basalts, which formed during Pangaea rifting when the Almopias ocean began forming. G17 may indicate a flood basalt having MORB-type REE patterns.

Therefore, in agreement with previous published views [7, 8], the Triassic formations of the Glossa peninsula are considered to belong to the pre-Upper Triassic basement of Skopelos, on the grounds of topographic/stratigraphic superposition of the Triassic dolomites. The petrographic and geochemical data correlate well with the Middle Triassic basement of Evvoia, and the Glossa series bears little resemblance to the Eohellenic complex, which in Evvoia, consists of serpentinised peridotite, gabbro, basalt and radiolarian cherts.

### The shear zone

The mineral assemblages are thought to indicate that retrograde phyllic metamorphism (decomposition of plagioclase) and pro-grade greenschist facies metamorphism (chlorite) had alternated with brittle-ductile deformation phases. Structurally, tectonic fabrics recur at progressively smaller scales (e.g. anastomising, lenticular shear planes at metre and microscopic scales), reminiscent of reiterating fractal- and unpredictable random chaotic-patterns [54, 55]. Retrograde, metasomatic sericite and secondary quartz together with opaque matter [iron (Fe)-oxides] is older than syn-tectonic pro-grade-metamorphic chlorite, epidote and chloritoid. It is supposed that sericitisation began in the ocean-floor realm resulting from metasomatic hydration reactions (phyllic alteration) during hydrothermal decomposition of the feldspars [30], and within a low temperature range below 300°C [30, 56]. Later, greenschist-facies temperatures (350°C) were probably reached during tectonic transport as a result of friction heat. The early quartz that formed as a product of sericitisation of feldspars has typical ragged outlines and fluid inclusions, as opposed to syntectonic idioblastic quartz that is partly or completely cleansed of impurities. The hydrothermal environment apparently led to the complete destruction of olivine, which broke down to magnetite or hematite [57]. The greenschist-facies mineralisation succeeded phyllic alteration. The greenschist metamorphic processes described did not affect the carbonate substrates over which the shear zone rocks advanced.

### Pseudotachylite

The fluxion structured fabric of porphyroclast-laden opaque matter, which is pervasively present in laminae of the shear zone rocks, indicates that the opaque matter was a more or less viscose fluid medium interpreted here as pseudotachylite [26, 58]. Normal optical investigations with the polarising microscope could not determine whether glass had been or still is present in the opaque matter and would require refined investigations. Although the shear-zone rocks, based on the mineral observations, only underwent low grade dynamic metamorphism in the temperature range of about 250°C to 350°C, it is nevertheless very possible that heat of friction in the slipping and sliding planes could have caused localised melting [26], which in the present case is supposed to have alternated with phases of cooling and solidification at different times and levels within the nappe as it advanced. These interpretations have been tentatively supported by microscope observations and discussions carried out together with Professor S. Heuss-Asbichler (MSc Mineralogy) and A. Huber (MSc Mineralogy), of the Department of Earth and Environmental Sciences, Ludwig-Maximilians University Munich.

The early ‘quartz’ porphyroclasts were products of the sericitisation of feldspar (see above) and typically contained abundant fluid inclusions, and very possibly were amorphous or cryptocrystalline, which would have been soluble at lower temperatures than crystalline quartz [59]. Local friction heating (> 350°C or more) of short duration is envisaged to have transformed shear laminae into a fluid medium that melted the outer fringes of the therein transported, very possibly, amorphous or cryptocrystalline-silica porphyroclasts (Fig. 4d).

### Geochemistry and Eu anomalies

Negative Eu anomalies are not common in MORB and are typically absent from OIB [46–49, 52, 60]. Therefore, the negative Eu anomalies of the study area present a paradox to what should be expected, because relict plagioclase laths occur in the basalt lithic grains and throughout the better preserved shear zone rocks, from which Eu enrichment should be expected [60, 61].

The observed plagioclases, however, for the most part, are sericitised ghosts from which, it is suggested, Eu was lost together with calcium (Ca) during sericitisation [30], which would explain the negative anomalies. Therefore, it is plausible that the original magma was of MORB origin, in which subsequent ocean-floor metasomatic removal of Eu occurred. From the bio-geological point of view, the occurrences of radiolarian chert together with the observed litho-grains of basalt indicate that most of the shear-zone rocks were derived from the ocean floor and not from an island arc source. Nevertheless, the discrimination diagrams (Fig. 8b) show that numerous samples have IAB affinities, which implies that the rocks of the shear-zone may have originated from two sources (see below).

### The geotectonic evolution of the study area

**Figure 9:**
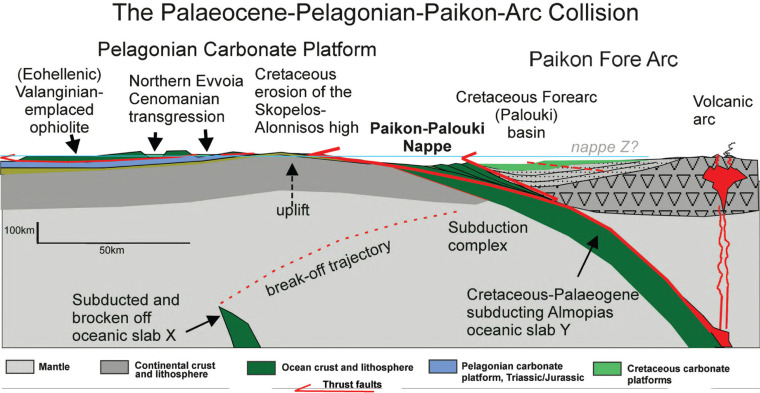
Cartoon of the Palaeocene, Pelagonian–Paikon Arc collision (approximate scale). The Eohellenic ophiolite (Almopias oceanic crust) was obducted westwards onto the eastern, continental Pelagonian plate, and the eastern Almopias plate subducted eastwards beneath the Paikon Arc. Two subductions are indicated: slab x (= the leading oceanic edge of the Pelagonia), and slab y (= the eastern half of the Almopias plate). Slab x is supposed to have broken off during the Early Cretaceous, thereby liberating the relatively buoyant eastern-Pelagonia and initiating uplift and erosion on the Sporades. Continued convergence finally caused the collision of eastern Pelagonia with the Cretaceous, carbonate-forearc basin, whereby the Paikon arc acted as a backstop. The Cretaceous carbonate platform overthrust eastern Pelagonia (Sporades) together with scraped off ocean floor m¨¦lange (shear zone formation) (see text).

The Cretaceous carbonates of the investigated islands do not have a sedimentary substrate; they are in allochthonous tectonic contact with shear-zone rocks of oceanic-floor origin (radiolarites and basalt), which is considered here to have been the glide horizon upon which the Cretaceous carbonate platform of the Paikon–Palouki nappe was transported. From the biostratigraphic evidence (see above), the Cretaceous carbonate platform was a shallow marine, reefal facies that evolved in early Late Cretaceous to Maastrichtian/Palaeocene time, so that the Paikon–Palouki nappe cannot have been transported before Early Palaeocene time. This leads to the question, where did the shallow marine, reefal, carbonate platform evolve? From the composition of the shear-zone substrate, it may have been an area of uplifted ocean-floor rocks, for example, Eohellenic metabasites, over which the carbonate platform could have accumulated *in situ*, (analogous to Evvoia, see [19]). An alternative is that the Cretaceous carbonates are of Vardar zone origin in analogy to a similar Cretaceous carbonate platform reported to have developed in the Paikon subzone [62]. We envisage here a plate-tectonic, platform-setting [63], inferring that the Cretaceous carbonate platform evolved in the forearc basin of the Vardar–Paikon subzone and subsequently was transported, some hundreds or a thousand kilometres, over ocean floor rocks (Fig. 9). Present day forearc basins do not harbour carbonate platforms thicker than about 100 m [64], which is not surprising considering the climatic and sea-level fluctuations of the Pleistocene. However, the supposed forearc basin proposed here, had all of Cretaceous time to accumulate and there are examples of ancient forearc basins in which thick reefal carbonate platforms had evolved [64]. The present model (Fig. 9) suggests that the carbonate platform of the forearc basin gradually overthrust the simultaneously subsiding Sporades, together with scrapped-off MORB and IAB mélange as the Almopias oceanic plate was subducting and the eastern Pelagonian plate collided with the Paikon–Paeonian back-stop. [The indications of high temperature (450°C) and pressure reported by Porkoláb et al. [28] are considered in this contribution to have been caused by tectonic compression of the shear zone rocks between the subducting Almopias plate and the arc back-stop.]

It has been pointed out [6], that an additional overthrust, which no longer exists, presumably having been removed by erosion, may have caused the metamorphism of Cretaceous marbles. However, considering that the marbles occur tectonically interleaved with recrystallised fossiliferous limestone, it is plausible that imbrication-faulting within the Paikon–Palouki nappe caused low-grade metamorphism with differing intensities as a result of frictional heating. Recrystallisation of limestone can be enhanced at temperatures even below 200°C [65–67]. This alternative hypothesis would eliminate the theoretical necessity of a ‘Paeonian nappe Z’ (Figs 2a and 9). However, this model does not describe the final collisions between Paikon, Paeonia and the European plate, and the closure of Neotethys.

### Subductions of two slabs of oceanic lithosphere

**Figure 10:**
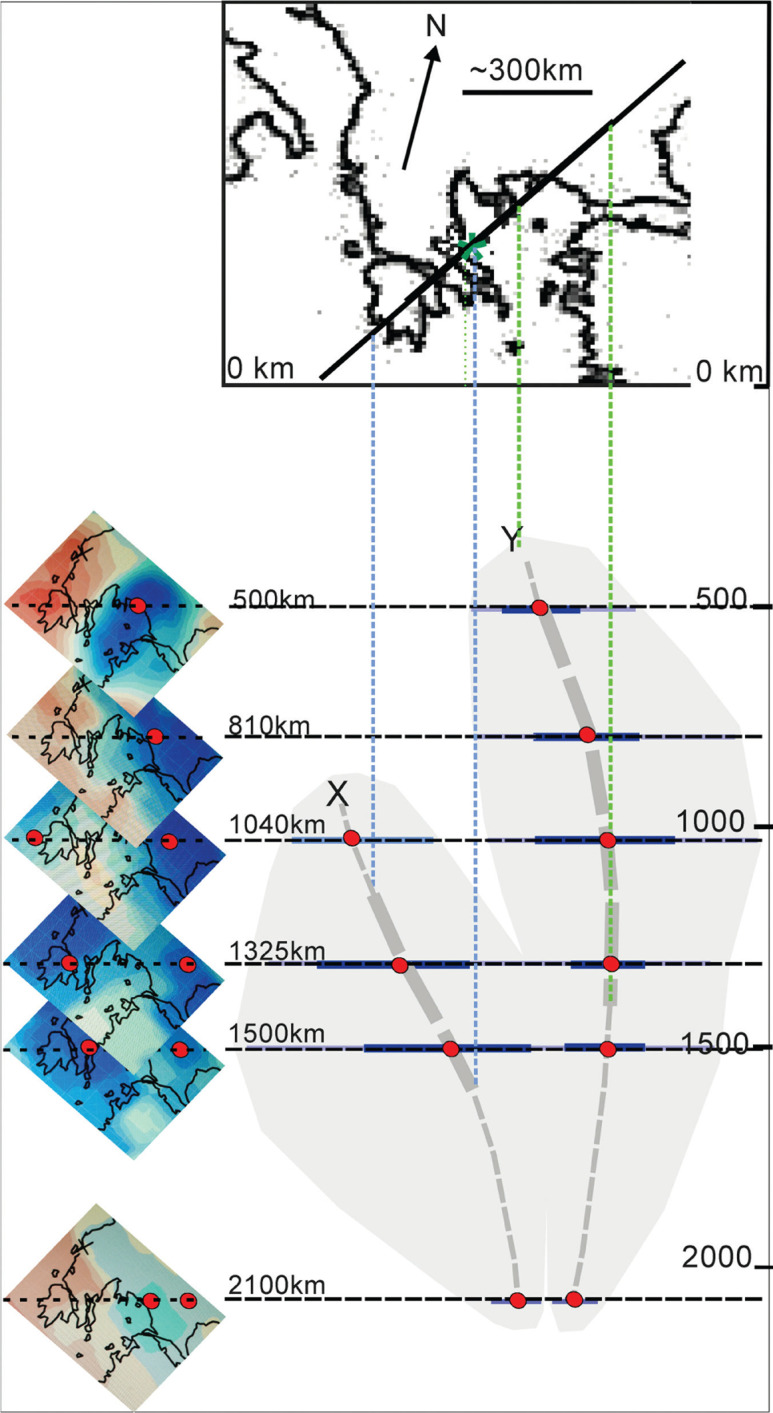
Seismic tomographic interpretation of positive seismic velocity anomalies beneath a NE–SW section through the mantle below the study area (green asterisk), constructed from BSE-images of the Bijwaard et al. [68] Model, ascertained from Hafkenscheid [69]. This model is based on enlargements of the Aegean region from the BSE-model [68]. The images, at the left show the trace of the NE–SW vertical section through the six levels of the mantle (500–810–1040–1325–1500–2100 km) below the study area; red dot at location of strongest signal. The grey cloud at the right depicts the positive seismic velocity anomalies in the cross section through the mantle, interpolated from one BSE level to the next, with the strongest signals at the centre of the cloud (red dots). The heavy lines are supposed to represent the central portions of relict oceanic slabs of which there are apparently two: X and Y. Slab X must have broken off before slab Y. The upper-most tomographic perturbations of slab X are at about 900 km depth, which is the depth of subsidence since the break-off; slab Y reached a depth of about 400 km presumably after the collision of Pelagonia with the Paikon arc. Both slabs, X and Y, sank down nearly to the same level, at about 2100 km, which infers that they had subducted more or less simultaneously, or intermittently, and reached approximately the same depth.Extra information *UCL Open: Environment* is an open scholarship publication, all previous versions and open peer review reports can be found online in the *UCL Open: Environment Preprint* server at ucl.scienceopen.com

Our model (Fig. 9) emphasises that two separate subductions took place, represented by slab X and slab Y in Fig. 2b. Slab X is the oceanic leading edge of the continental Pelagonian plate and slab Y is the subducting eastern Almopias oceanic plate. In order to test the model, shown in Fig. 9, a tomographic reconstruction, following the Bijwaard-Spakman, Engdahl (BSE) model [68] was used to construct a NE–SW vertical section through the mantle below the study of the Aegean area (Fig. 10). The patterns of the seismic anomalies in the Aegean region below the study area (in the BSE models) appear to substantiate that two slabs (X and Y) were indeed subducted, and they best fit the envisaged surface geology.

In a corresponding vertical section through Crete and the Bosphorus, the BSE tomographic model of Bijwaard et al. (fig. A8 in [69]; section 15 in [68]; and 15 in fig. 5 of Hafenschied et al., 2006) shows two anomalies, which are interpreted in three different models depicting the closure of the Neotethys. From the point of view of our study we interpret remnants of two subducted slabs: one between 500 km and 1800 km depth, the other, between the near surface to 1500–2000 km depth.

It is realised that many variables and many unknown factors make tomographic reconstructions highly complex [68–70], including for example the velocities of the sinking slabs, the drift rates and direction of the European plate, the thickening of the slabs as they sink, competing forces of buoyancy verses viscous drag [71]. Further problems concern the *break-off times* of the sinking slabs, which are only roughly imaged, but are theoretically known to have occurred, based on the surface geology and the tomographic models. From Fig. 10, the leading edge of the slab X sank 900 km since breaking off, probably after the Valanginian time because the break-off is supposed to have triggered extreme erosion on Skopelos and Alonnisos. Slab X continued to sink while contemporaneously subduction was taking place beneath the Paikon arc. The emplacement of the Paikon–Palouki nappe occurred during the Early Palaeocene as a result of the collision of eastern Pelagonia with the Paikon arc. Since its break-off, slab Y has sunk about 400 km (Fig. 10), and, from Fig. 10, the leading edges of both slabs presently have reached the depth of about 2100 km.

Using the available variables, it is not possible to arrive at a tenable time schedule of subduction. The rates of subsidence of the oceanic slabs are reported to range from about 3 cm/year in the upper mantle to about 1 cm/year in the lower mantle [70]. These rates are much too high for the model we envisage. However, the sinking rates might be much lower below 300–500 km, in the lower mantle, eventually approaching zero (see [72, 73], and others in [71]).

In spite of these insecurities, a few estimates are attempted here. The depth of slab-subsidence since the break-off is known (from the BSE model) and the times of the break-off (from the surface geology) are Post-Valanginian (∼130 Ma) for slab X, and Late Palaeocene (∼58 Ma) for slab Y. Using an ‘*average’* subsidence rate of 0.68 cm/year, we arrive at a break-off date of slab X [900 km/0.68 cm ∼132 Ma (using identical units)] Hauterivian/Barremian, and the break-off of slab Y (400 km/0.68 cm ∼58 Ma) Late Palaeocene.

Although the above model is speculative, the BSE model of Fig. 10 supplies enough information to estimate the one-time width of the Almopias Ocean by adding together the lengths of the subducted slabs, with the result that the Almopias Ocean had a width of about 2500–3000 km. This does not mean that there had been an ocean of this width at any one time because subduction and obduction could have taken place simultaneously together with an actively spreading mid oceanic ridge.

The *Paikon-Palouki nappe* which is assumed to have covered the islands of the Sporades did not reach Evvoia. (Field work has not been carried out on Skiathos or on Skyros (compare [74].) Palaeocene/Eocene uplift and normal faulting most likely occurred on Alonnisos and Skopelos around the same time as on Evvoia [3]. Post collision faulting in the Aegean area, north and north-west of the study area, is described in [41].

## Conclusions

The pre-Upper Triassic basement of Evvoia and the successions of rocks exposed in the Glossa area of Skopelos have been shown to correlate well. They occur stratigraphically below Pelagonian Upper Triassic dolomite separated by an intra-formational fault zone. The basement of Evvoia and Skopelos is assumed to extend to the Pelagonian/Vardar border (or Sava suture, [5]) eastwards beyond the island of Gioura. The metabasites of the Glossa basement have been inferred to have been rift basalts that formed during the Permo-Triassic break-up of Pangaea.

Typical Pelagonian platform carbonates are encountered on all the studied islands. The stratigraphic succession on Evvoia is almost complete, whereas the Eohellenic ophiolite and the Jurassic has been eroded from Skopelos, and on Alonnisos the Eohellenic ophiolite has been more or less completely eroded. The Early Cretaceous period of intensive erosion is envisaged to have been caused by the buoyancy-rebound of eastern Pelagonia after the leading oceanic edge of the Pelagonian plate had broken off and sank.

While erosion was taking place on Skopelos and Alonnisos, the eastern side of the Almopias oceanic plate continued subducting beneath the Paikon island arc and simultaneously, Cretaceous carbonate platforms evolved over the eroded ophiolite in Evvoia, and evolved in the forearc basin of the Paikon island arc complex.

Ultimately, eastern Pelagonia collided with the Paikon arc whereby the Cretaceous/Palaeocene forearc platform was overthrust westwards, initially over ocean floor rocks, before it was emplaced over the Pelagonian carbonates of Alonnisos and Skopelos and neighbouring islands. Documenting the emplacement of this Paikon–Palouki nappe is a dynamically metamorphosed shear-zone formation of oceanic and carbonate rocks, characterised by fluxion structured cataclasites, mylonites, phyllonites and pseudotachylites.

We calculate a plausible width of the Almopias ocean to be a few 1000 km. The final stages of the closure of the Almopias ocean took place during two episodes: first (Late Jurassic-Early Cretaceous), as the Eohellenic ophiolite (west Almopias) was obducted westwards and the leading oceanic edge of the Pelagonian plate was being subducted, and second (Early Palaeocene), as eastern Almopias subducted beneath the Paikon island arc. It is suggested that the relicts of two subducted slabs of Almopias oceanic lithosphere can be seen in BSE seismic-tomographic images of the mantle beneath the Aegean region.

Future research would be rewarding in the following fields: further correlations between the Pelagonian basement of NW Evvoia and NW Skopelos; investigations of the shear-zone formation in respect to a full-fledged fabric analysis of the mylonites and possible pseudotachylites; possible radiolarian extractions and dating; better seismic tomographic models; investigation and reconstruction of the Cretaceous carbonate platform; and further analysis of post collision deposition and neo-tectonics.

## Data Availability

All data generated or analysed during this study are included in this published article (and its supplementary information files).
